# Association between the arm circumference and non-alcoholic fatty liver disease in American children and adolescence: a population-based analysis

**DOI:** 10.3389/fpubh.2024.1323795

**Published:** 2024-05-27

**Authors:** Xiaolu Weng, Jing Xu, Shouxing Yang

**Affiliations:** ^1^Department of Endocrinology, The Second Affiliated Hospital and Yuying Children's Hospital of Wenzhou Medical University, Wenzhou, China; ^2^Department of Gastroenterology, The Second Affiliated Hospital and Yuying Children's Hospital of Wenzhou Medical University, Wenzhou, China

**Keywords:** arm circumference, non-alcoholic fatty liver disease, children, adolescence, liver steatosis

## Abstract

**Background:**

The arm circumference (AC) has been used as an important tool to access the risk of non-alcoholic fatty liver disease (NAFLD) in adults. However, the association between AC and NAFLD in children and adolescence remains unclear. This study aims to explore the relationship between AC and NAFLD in American children and adolescence.

**Methods:**

2017–2020 National Health and Nutrition Examination Survey (NHANES) was used to carry out the cross-sectional study. The association between AC and the risk of NAFLD, and liver steatosis was analyzed using weighted multivariable logistic regression and multivariate linear regression. Additionally, a two-part linear regression model was used to identify threshold effects in this study. Subgroup analysis, interaction tests and receiver operating characteristic (ROC) curve analysis were also carried out.

**Results:**

A total of 1,559 children and adolescence aged 12–18 years old were included, and the prevalence of NAFLD was 27.3%. AC was positively correlated with the risk of NAFLD (OR = 1.25, 95% CI: 1.19, 1.32) and liver steatosis (*β* = 4.41, 95% CI: 3.72, 5.09). Subgroup analysis stratified by age and race showed a consistent positive correlation. A non-linear relationship and saturation effect between AC and NAFLD risk were identified, with an S shaped curve and an inflection point at 34.5 cm. Area under the ROC of AC to NAFLD was 0.812, with the sensitivity of 67.6%, the specificity of 83.8% and the cutoff value of 31.7 cm.

**Conclusion:**

Our study shows that AC is independently correlated with an increased risk of NAFLD and the severity of liver steatosis in American children and adolescence.

## Introduction

Nonalcoholic fatty liver disease (NAFLD) refers to a severe medical condition characterized by the degeneration of more than 5% liver cells, which is not caused by drinking alcohol or any specific factors. As a result, fat accumulates in the liver over time, thus resulting in chronic liver damage (1). The prevalence of NAFLD has reached alarming levels worldwide, exceeding 25%. Due to the increase of obesity rate and lifestyle changes, it is expected to become the most common liver disease in the next decade (2). Children and adolescence are not immune to this condition, and the current global incidence ranges from 3 to 10% among this population (3). For these young individuals, NAFLD is a significant risk factor for developing severe cardiovascular diseases in adulthood, including chronic kidney disease, diabetes mellitus and stroke (4). Therefore, in order to ensure the long-term health of children and adolescence and reduce the burden on the medical system, it is imperative to give priority to the early prevention and intervention strategies of NAFLD in children and adolescence.

NAFLD is a metabolic syndrome known for its manifestation in the hepatic area, of which progressive form, NASH, poses an increased risk of liver cancer, end-stage liver disease, and cirrhosis (5). Pediatric NAFLD is associated with the risk of diabetes mellitus (DM), metabolic syndrome, and cardiovascular disease (CVD) (6). The diagnosis of NAFLD in children and adolescence can be challenging as they often show no symptoms. Liver biopsy, the gold standard for NAFLD diagnosis, is invasive and not readily accepted by children. Although liver ultrasonography is a feasible and noninvasive diagnostic method, not all children undergo this procedure due to parental concerns over exposing them to medical examinations for a seemingly asymptomatic condition (7). Therefore, it is crucial to identify valuable indicators of NAFLD to enable early detection and prevention of further progression.

Arm circumference (AC) is a conveniently accessible, straightforward, and cost-effective marker, and some studies have shown that it can be used as a new index to evaluate metabolic syndrome, insulin resistance, and NAFLD in adults (8–10). However, it is not clear whether AC is related to NAFLD in childhood or adolescence. Consequently, this study aims to investigate the correlation between AC and NAFLD in children and adolescence.

## Materials and methods

### Study population

In this study, the data analyzed was from the National Health and Nutrition Examination Survey (NHANES) (2017–2020), which belongs to a complex, stratified, and multi-stage probability sample of the uninstituted population in the United States. These cross-sectional surveys were carried out by National Center for Health Statistics (NCHS). Details of NHANES methods can be found at www.cdc.gov/nchs/NHANEs/.

**Figure 1:**
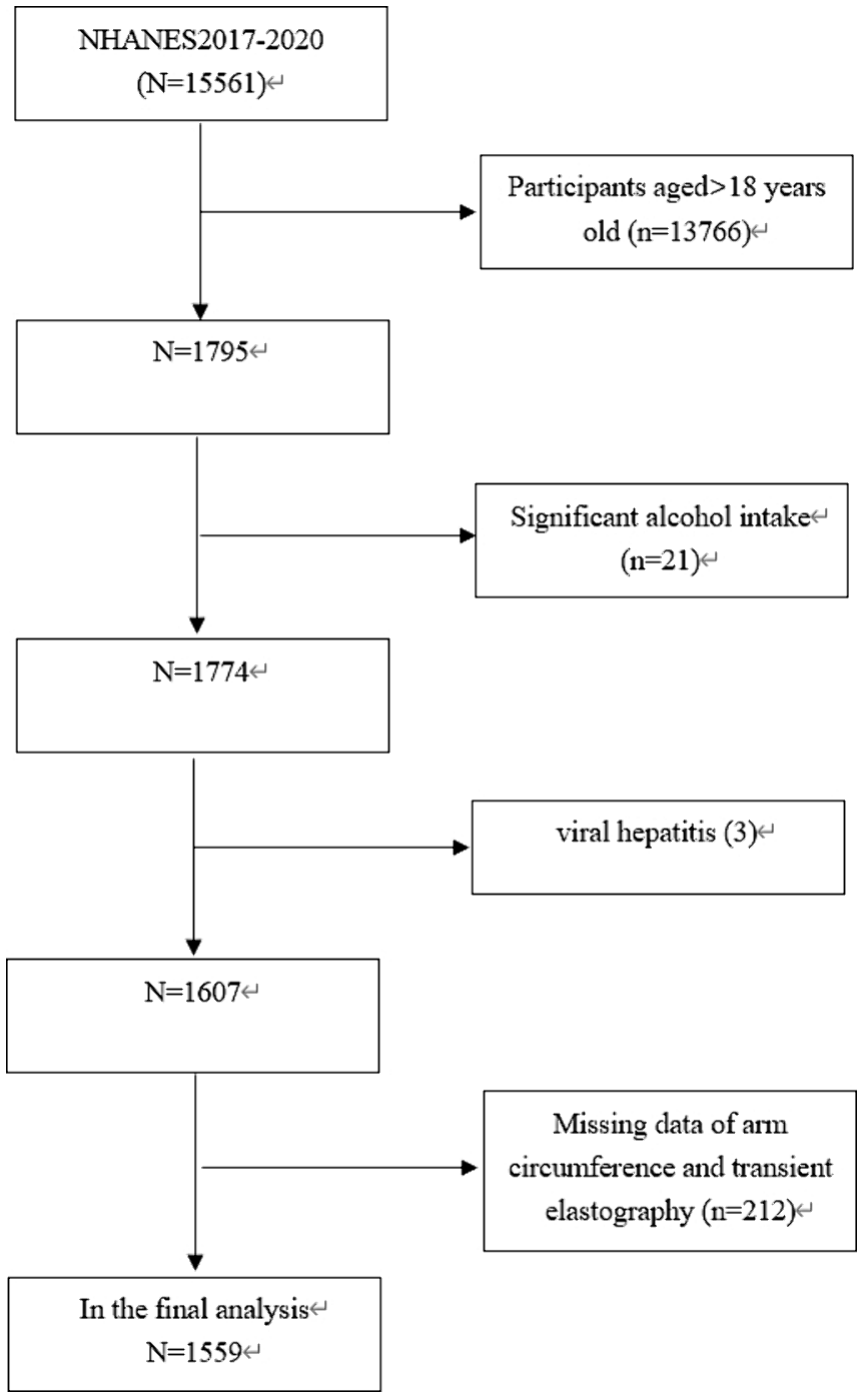
Flowchart of the sample selection from the 2017–2020 NHANES.

The study focused exclusively on participants who were under the age of 18 (*n* = 1,795). Twenty one individuals who reported excessive alcohol consumption (more than 14 drinks per week for females and more than 21 drinks per week for males) were excluded (11). In addition, 215 participants were eliminated due to viral hepatitis (3), missing data on AC (*n* = 164) or transient elastography (TE) data (*n* = 48). Consequently, our final analysis involved 1,559 participants aged 12–18 years old (Figure 1).

The NCHS Ethics Review Board approved the implementation of NHANES, and all participants provided written informed consent (12).

### VCTE

In the NHANES database, the FibroScan 502 V2 Touch (Echosens) was used to measure liver VCTE in participants from 2017 to 2020. This particular device is well-suited for studying NAFLD, a condition characterized by fatty liver disease. In order to assess liver fibrosis and steatosis in patients with fatty liver disease, validated parameters such as liver stiffness measurements (LSMs) and controlled attenuation parameter (CAP) were utilized (13, 14). For VCTE outcomes to be considered valid, certain criteria were followed. This included acquiring a minimum of 10 LSMs after fasting for at least 3 h, with an interquartile range (IQR) range/median of less than 30% (15). It is used to determine CA*p* values ≥288 dB/m were NAFLD status (16).

### Study variables

AC was assessed while the participant maintained an upright position with their arms relaxed at their sides. A measuring tape was used to encircle the right upper arm at a point perpendicular to its length (17). The measurement results were accurate to millimeter. In addition, the following covariates were included: age, alanine aminotransferase (ALT), gender, race, body mass index (BMI), moderate activities, weight, albumin, aspartate transaminase (AST), total cholesterol (TC), gamma-glutamyl transpeptidase (GGT), creatinine, alkaline phosphatase (ALP), blood urea nitrogen, low-density lipoprotein cholesterol (LDL-C), glycohemoglobin (HbA1c), high-density lipoprotein cholesterol (HDL-C), total bilirubin, and triglyceride (TG). Details of NAFLD and AC measurement process and other covariate acquisition process were available at www.cdc.gov/nchs/nhanes/.

### Statistical analysis

In this analysis, continuous data were expressed as weighted mean ± standard deviation (SD), while categorical variables were expressed as weighted proportions. The study participants were divided into four groups or quartiles based on their AC levels. In order to evaluate the differences between each group, the weighted χ^2^-test was used for categorical variables, and a weighted linear regression model was applied for continuous variables. In order to investigate the association between AC and NAFLD status, a weighted multivariate logistic regression model was adopted. Furthermore, a weighted multivariate linear regression analysis was conducted to explore the connection between AC and liver steatosis, assessed by liver CAP. Subgroup analyses were performed to examine the potential impacts of gender, BMI, age, and race on the relationship between AC and NAFLD. In order to identify any potential nonlinear relationship between AC and NAFLD probabilities, smooth curve fitting and generalized additive model were used. Finally, the diagnostic effectiveness of AC in detecting NAFLD was evaluated using ROC curve studies. The statistical analysis was conducted using EmpowerStats software^1^ and R,^2^ with significance determined at a *p*-value of <0.05.

## Results

### Baseline characteristics of participants

A total of 1,559 participants aged 12–18 years old were included in this study, the prevalence of NAFLD was 27.3%. The weighted population characteristics of participants by serum AC quartiles (Q1: <25.4 cm; Q2: 25.4–28.4 cm; Q3: 28.4–32.6 cm; Q4: >32.6 cm) were shown in Table 1. Compared with the bottom quartile, those in the top AC quartile were more likely to be older adult and male, with a higher proportion of Non-Hispanic White, a higher prevalence of NAFLD, and the increased levels of weight, BMI, ALT, GGT, creatinine, uric acid, glycohemoglobin, TC, TG, LDL-C, LSM and CAP. In contrast, the levels of albumin, ALP, total bilirubin and HDL-C were lower (*p* < 0.05).

**Table 1:** Weighted characteristics of the study population based on AC quartiles.

Characteristic	Q1	Q2	Q3	Q4	*P*-value
Number	388	383	394	394	
Age, year	14.02 ± 1.93	14.98 ± 1.89	15.24 ± 1.89	15.54 ± 1.86	<0.001
NAFLD, %					<0.001
Yes	7.7	11.5	26.6	62.7	
No	92.3	88.5	73.4	37.3	
Sex, %					0.020
Male	49.7	47.8	56.6	56.6	
Female	50.3	52.2	43.4	43.4	
Race, %					0.049
Mexican American	15.2	12.0	18.0	17.8	
Other Hispanic	10.8	9.1	8.4	5.6	
Non-Hispanic White	31.2	31.1	29.7	33.2	
Non-Hispanic Black	20.9	27.9	25.4	26.6	
Other race	21.9	19.8	18.5	16.8	
Moderate activities, %					0.116
Yes	62.1	64.3	43.6	47.3	
No	37.9	35.7	56.4	52.7	
Weight, kg	46.99 ± 6.96	58.25 ± 5.68	69.65 ± 7.77	94.80 ± 19.18	<0.001
BMI, kg/m^2^	18.37 ± 1.76	21.64 ± 1.78	25.02 ± 2.68	33.30 ± 6.31	<0.001
AC, cm	23.1 ± 1.7	26.8 ± 0.8	30.4 ± 1.2	36.7 ± 3.9	<0.001
Albumin, g/dL	4.33 ± 0.28	4.29 ± 0.29	4.28 ± 0.31	4.18 ± 0.35	<0.001
ALT, U/L	12.5 ± 6.0	13.9 ± 10.0	16.1 ± 9.6	22.1 ± 15.5	<0.001
GGT, IU/L	11.8 ± 3.7	14.8 ± 15.8	15.1 ± 8.6	19.5 ± 11.4	<0.001
ALP, IU/L	193.9 ± 105.7	146.2 ± 100.7	138.1 ± 84.6	133.5 ± 74.4	<0.001
Total bilirubin, μmol/L	7.8 ± 5.7	8.3 ± 5.6	8.2 ± 5.9	6.8 ± 4.6	0.016
Creatinine, mmol/L	55.2 ± 12.2	63.0 ± 13.6	66.7 ± 16.0	66.2 ± 14.0	<0.001
Uric acid, umol/L	263.5 ± 65.4	285.6 ± 69.7	306.1 ± 69.3	340.9 ± 78.6	<0.001
HbA_1_c, %	5.3 ± 0.4	5.3 ± 0.3	5.3 ± 0.6	5.4 ± 0.7	0.009
TC, mmol/L, mmol/L	3.88 ± 0.70	3.97 ± 0.72	4.00 ± 0.81	4.06 ± 0.79	0.002
TG, mmol/L	0.91 ± 0.44	0.90 ± 0.44	1.06 ± 0.62	1.29 ± 0.87	<0.001
LDL-C, mmol/L	2.08 ± 0.57	2.21 ± 0.59	2.27 ± 0.76	2.46 ± 0.67	<0.001
HDL-C, mmol/L	1.46 ± 0.31	1.43 ± 0.30	1.30 ± 0.27	1.17 ± 0.24	<0.001
LSM, kPa	4.77 ± 1.28	5.00 ± 2.69	5.07 ± 1.73	5.71 ± 3.04	<0.001
CAP, dB/m	192.6 ± 40.0	203.3 ± 38.7	223.4 ± 46.5	266.7 ± 54.8	<0.001

Values are mean ±SD or number (%). *P* < 0.05 was deemed significant. BMI, body mass index; AC, arm circumference; HbA1c, glycosylated hemoglobin; TC, total cholesterol; TG, triglyceride; HDL-c, High density lipoprotein cholesterol; LDL-c, Low density lipoprotein cholesterol; ALT, alanine aminotransferase; GGT, gamma-glutamyl transpeptidase; ALP, alkaline phosphatase; LSM, liver stiffness measurements; CAP, controlled attenuation parameter.

### Correlation between AC and NAFLD risk

Three weighted multivariate regression models were constructed between NAFLD prevalence and AC (Table 2). In the unadjusted model, AC was positively correlated with NAFLD probabilities [OR = 1.29, 95% CI: (1.25, 1.32)]. After adjusting for gender, race, age (model 2), Moderate activities, BMI, albumin, uric acid, ALT, GGT, ALP, uric acid, total bilirubin, creatinine, TC, TG, HDL-C and glycohemoglobin (model 3), this positive correlation remained in Model 2 [OR = 1.30, 95% CI: (1.26, 1.34)] and Model 3 [OR = 1.25, 95% CI: (1.19, 1.32)]. Moreover, compared with the lowest level of AC (Q1) in model 3 (P for trend <0.001), the NAFLD risk of subjects in quartiles 2, 3 and 4 increased by 0.99, 3.97 and 9.42, respectively. This result indicated that children and adolescence with elevated AC are more likely to develop NAFLD than those with decreased AC.

**Table 2:** Association between AC and NAFLD status in logistic regression analysis.

	Model 1 OR (95% CI) *P*-value	Model 2 OR (95% CI) *P*-value	Model 3 OR (95% CI) *P*-value
AC	1.29 (1.25, 1.32), <0.001	1.30 (1.26, 1.34), <0.001	1.25 (1.19, 1.32), <0.001
AC (Quartile)			
Q1	Reference	Reference	Reference
Q2	1.55 (0.95, 2.52), 0.078	1.71 (1.04, 2.80), 0.034	1.99 (1.13, 3.52), 0.017
Q3	4.34 (2.81, 6.69), <0.001	4.77 (3.05, 7.45), <0.001	4.97 (2.90, 3.52), <0.001
Q4	20.05 (13.11, 30.66), <0.001	23.05 (14.76, 36.00), <0.001	10.42 (5.53, 19.63), <0.001
P for trend	<0.001	<0.001	<0.001

Model I, None covariates were adjusted; Model II, gender, age and race were adjusted; Model III, gender, age, race, Moderate activities, BMI, albumin, uric acid, ALT, GGT, ALP, total bilirubin, creatinine, TC, TG, HDL-C and glycohemoglobin.

### Correlation between AC and severity of liver steatosis

**Figure 2:**
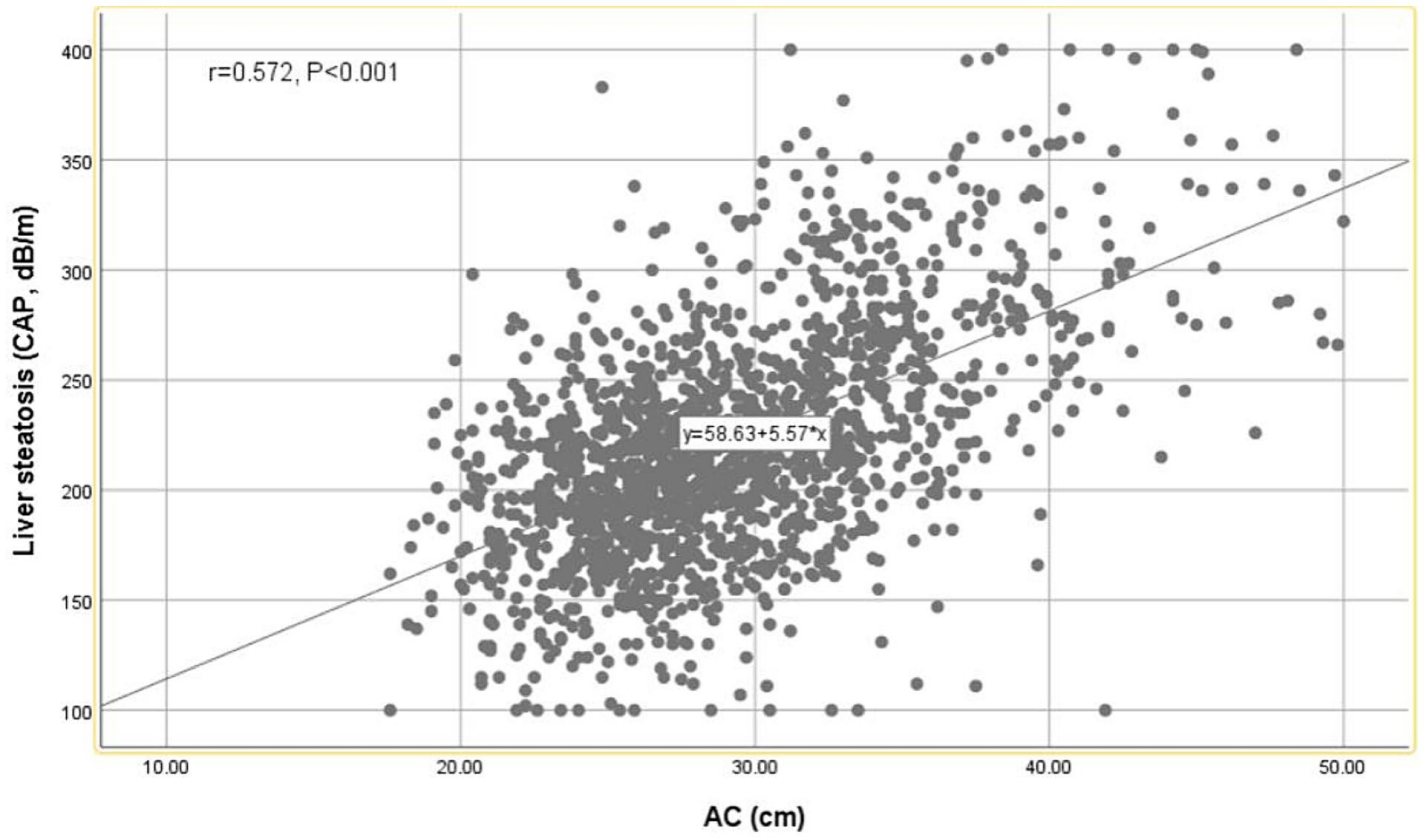
Scatter diagrams showing the correlation between the AC and CAP.

A strong positive correlation was observed between AC and CAP values using the Pearson correlation coefficient (*r* = 0.572, *p* < 0.001) (Figure 2). As shown in Table 3, AC is positively correlated with hepatic steatosis according to CAP values. In model 3, AC was dramatically and positively correlated with hepatic steatosis severity (*β* = 4.41, 95% CI: 3.72, 5.09) (*p* < 0.001).

**Table 3:** Associations between AC and CAP value in linear regression analysis.

	Model 1 β (95% CI) *P*-value	Model 2 β (95% CI) *P*-value	Model 3 β (95% CI) *P*-value
AC	5.57 (5.17, 5.97), <0.001	5.80 (5.39, 6.21), <0.001	4.41 (3.72, 5.09), <0.001
AC (Quartile)			
Q1	Reference	Reference	Reference
Q2	10.69 (4.25, 17.12), 0.001	12.12 (5.60, 18.64), <0.001	12.26 (5.53, 18.99), <0.001
Q3	30.81 (24.42, 37.20), <0.001	31.93 (25.39, 38.47), <0.001	28.81 (21.81, 35.81), <0.001
Q4	74.06 (67.67, 80.45), <0.001	75.55 (68.92, 82.17), <0.001	44.66 (35.20, 54.12), <0.001
P for trend	<0.001	<0.001	<0.001

Model I, None covariates were adjusted; Model II, gender, age and race were adjusted; Model III, gender (not adjusted for in the subgroup analyses), age, race, Moderate activities, BMI, albumin, uric acid, ALT, GGT, ALP, uric acid, total bilirubin, creatinine, TC, TG, HDL-C and glycohemoglobin.

### Subgroup analysis

**Figure 3:**
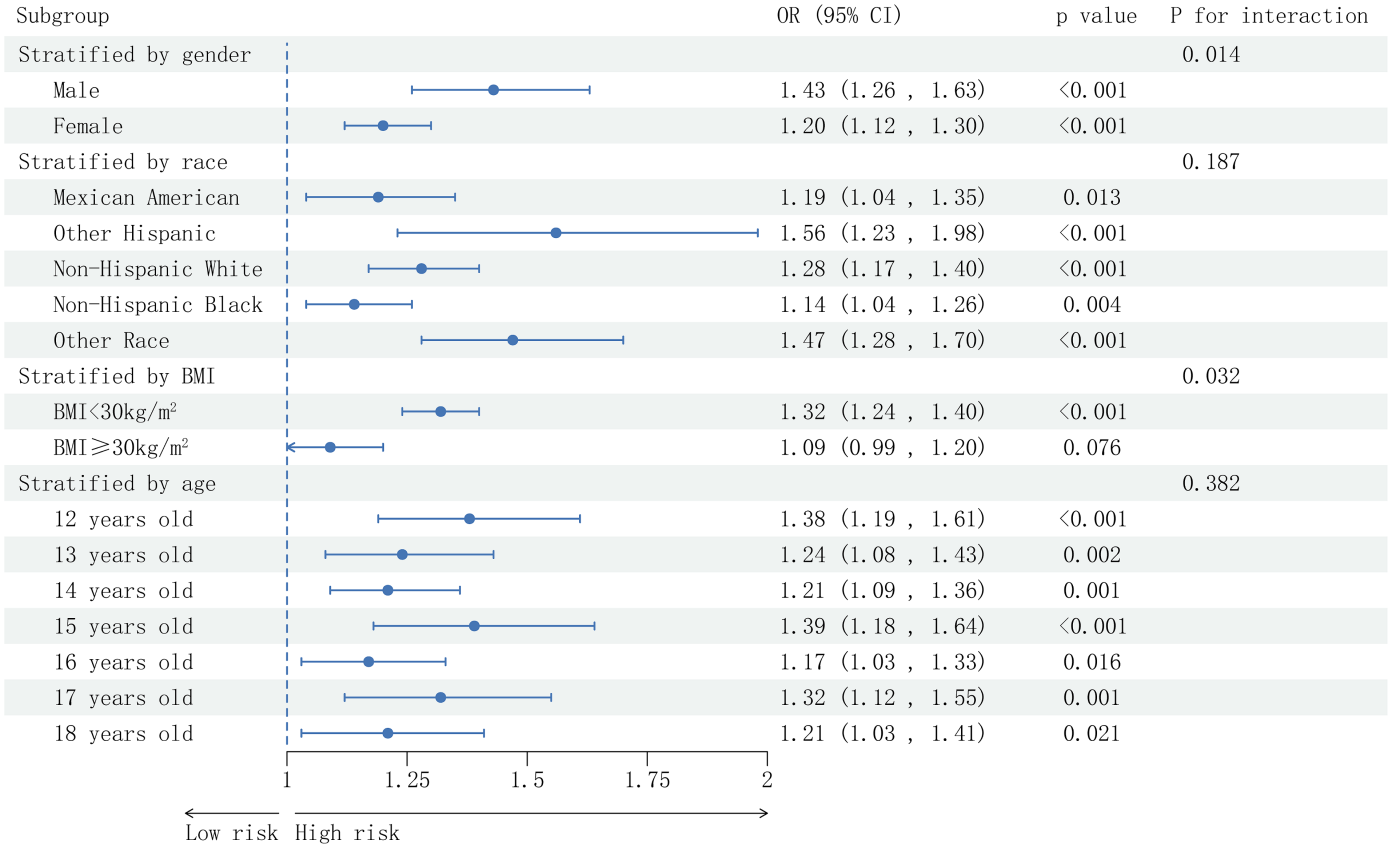
Subgroup analysis based on the analysis of multi-factor logistic regression for the association between the AC and prevalence of NAFLD. Gender, race, age, BMI (not adjusted for in the subgroup analyses), moderate activities, albumin, uric acid, ALT, GGT, ALP, uric acid, total bilirubin, creatinine, TC, TG, HDL-C and glycohemoglobin were adjusted.

A thorough subgroup analysis was conducted to assess the consistency of the association between AC and NAFLD risk in various demographic settings. As shown in Figure 3, the positive correlation between AC and NAFLD risk was not significantly affected by race and age (*p* > 0.05 for all interactions). The correlation between AC and NAFLD risk was stronger among male participants (OR = 1.43, P interaction = 0.014), among participants with BMI <30 kg/m^2^ (OR = 1.32, P interaction = 0.032). While in participants with BMI ≥ 30 kg/m2, this correlation did not reach statistical significance.

### Non-linearity and threshold effect analysis between AC and NAFLD

**Figure 4:**
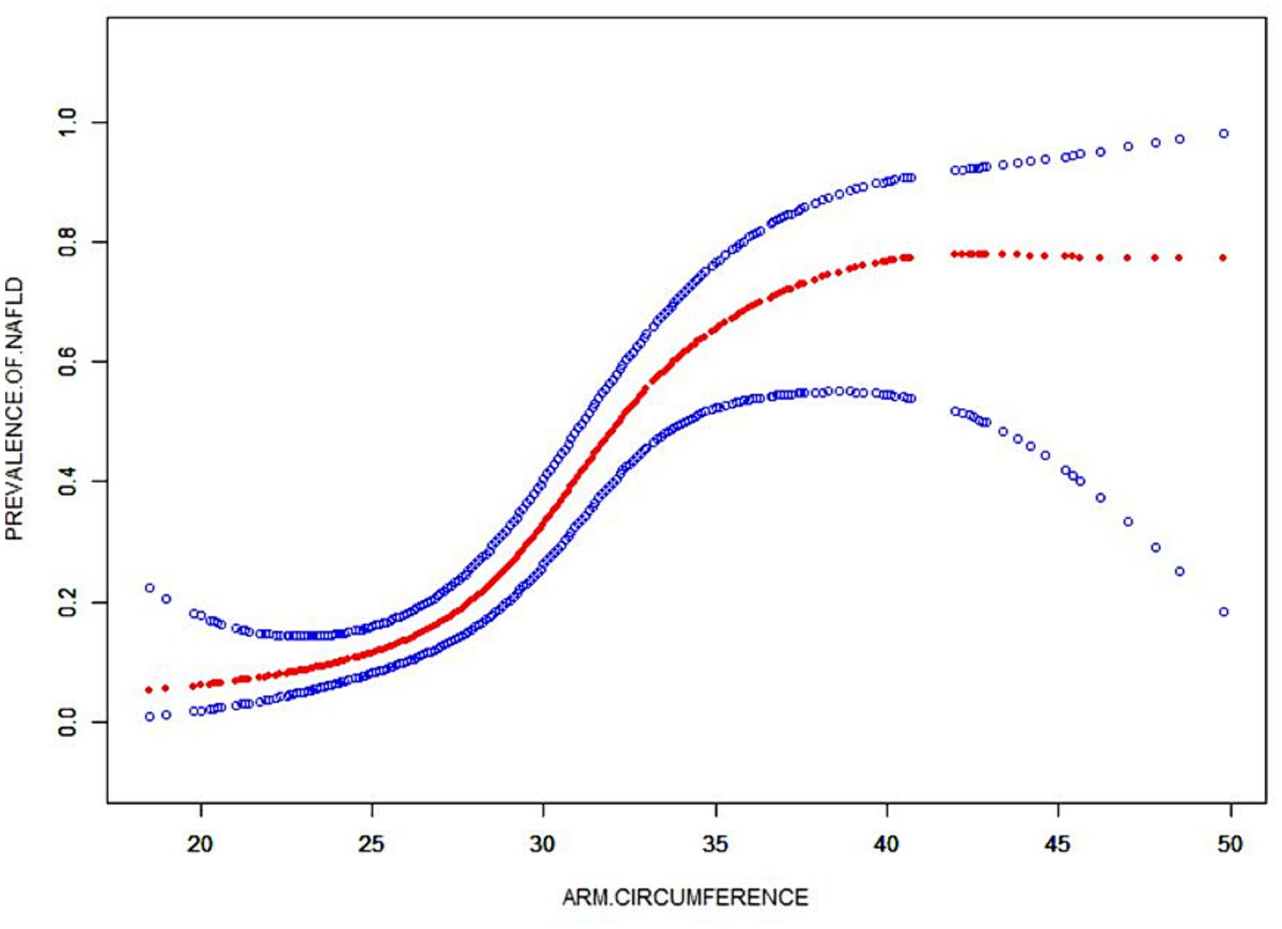
The smooth curve fit for the association between AC and prevalence of NAFLD. Solid redline represents the smooth curve fit between variables. Blue bands represent the 95% of confidence interval from the fit. Adjusted for: gender, age, race, moderate activities, BMI, albumin, uric acid, ALT, GGT, ALP, total bilirubin, creatinine, TC, TG, HDL-C and glycohemoglobin.

A smooth curve fitting technique was used to depict the non-linear relationship and saturation effect between AC and NAFLD status, as shown in Figure 4. Among all participants, the correlation between AC and NAFLD status exhibited an S-shaped curve, with inflection points observed at 34.5 cm (Table 4). When AC measurements were below 35.4 cm, there was a significant effect value of 1.324. However, when AC exceeded 35.4 cm, the effect values were not statistically significant.

**Table 4:** Threshold effect analysis of AC on NAFLD using the two-piecewise linear regression model.

Arm circumference	Adjusted OR (95% CI) *P*-value
Fitting by the standard linear model	1.252 (1.159, 1.353), <0.001
Fitting by the two-piecewise linear model	
Inflection point	34.5 cm
AC < 34.5 cm	1.324 (1.209, 1.449), <0.001
AC > 34.5 cm	1.040 (0.900, 1.203), 0.591
Log likelihood ratio	0.009

### ROC analysis

**Figure 5:**
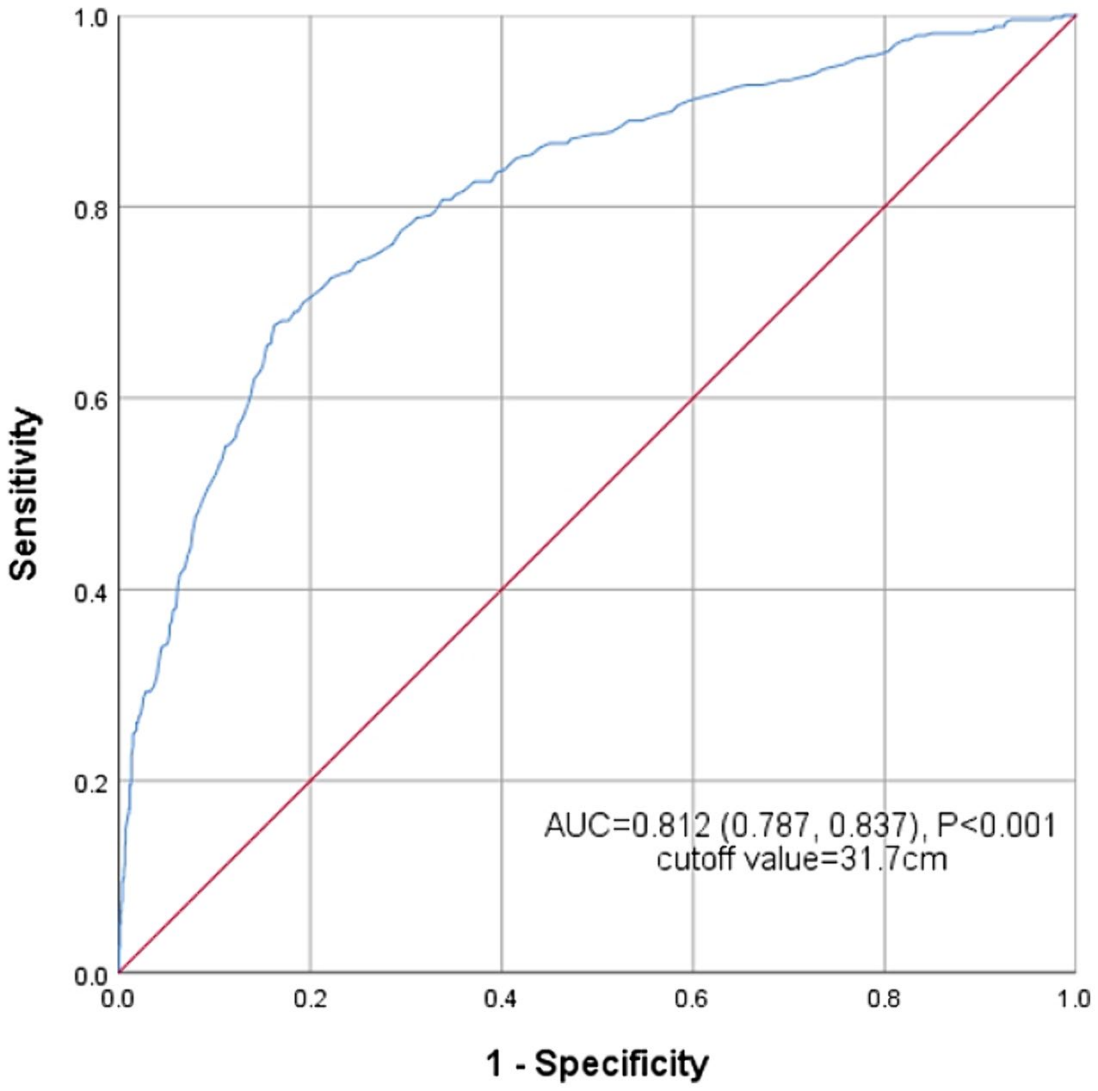
Receiver operating characteristic curves of AC to identify NAFLD.

Figure 5 shows the receiver operating characteristic (ROC) curve of the AC’s ability to identify NAFLD risk. The area under the curve (AUC) for AC in the ROC analysis was 0.812 (95% CI: 0.787–0.837), with the sensitivity of 67.6%, the specificity of 83.8% and the cutoff value of 31.7 cm.

## Discussion

Our extensive studies have revealed a positive correlation between AC and liver steatosis, as well as the risk of NAFLD among American children and adolescence. Through subgroup analysis and interaction assessment, it is found that this positive correlation remains consistent among subgroups of different ages and races, except for gender and BMI. It is worth noting that we have identified an intriguing S-shaped relationship between AC and NAFLD risk, with a distinct inflection point occurring at an AC measurement of 34.5 cm. Additionally, we have determined that AC displays a remarkable degree of sensitivity and specificity in the diagnosis of NAFLD. As far as we knowledge, it is the first study to investigate the association between AC and liver steatosis and the risk of NAFLD in American children and adolescence.

Previous studies have extensively explored the correlation between AC and NAFLD in adults (10, 18, 19). A notable study by Zou et al. examined this relationship specifically in adults aged 18 years old and above, and their findings illuminated a positive association between AC and the risk of metabolic dysfunction-associated fatty liver disease (MAFLD) (10). Similarly, Wang et al. conducted a study involving 2,397 adults diagnosed as MAFLD, and discovered a significant correlation between AC and liver steatosis as well as liver fibrosis (18). On the other hand, Ayonrinde et al. tracked the anthropometric characteristics of a cohort of individuals from birth, and observed that trajectories of AC, as an index of obesity, was closely related to NAFLD at the age of 17 beginning as early as 3 years old (19). However, there is no study on the diagnostic value of AC for NAFLD in children and adolescence. Our study results demonstrate that the AC is positively correlated with CAP values, which is consistent with a previous report (18). In addition, AC has high sensitivity and specificity in the diagnosis of NAFLD.

The results show that the correlation between AC and NAFLD risk exhibits a considerably stronger correlation in male individuals compared with their female individuals. This discrepancy might be attributed to the potential protective influence of estrogen in guarding against hepatic steatosis (20, 21). Investigations have revealed that estrogen may enhance insulin sensitivity, decrease liver *de novo* lipogenesis, minimize triglyceride buildup, and mitigate liver fibrosis and inflammation. As a result, the impact of estrogen on these mechanisms can explain the differing susceptibility to NAFLD between males and females (22–25). There is no observed correlation between AC and the risk of NAFLD among obese children and adolescence with a BMI > 30 kg/m2 or AC ≥ 35.4 cm. The influence of BMI, AC and NAFLD remains uncertain, but it can potentially be associated with the significant increase in skeletal muscle mass among obese children. Several studies have indicated that as the BMI, AC increases, there is a significant increase in skeletal muscle weight, skeletal muscle index, and limb fat. Furthermore, a higher muscle mass seems to be a negative correlation with the occurrence of NAFLD (26–28).

Our results also indicate that through further threshold effect analysis, there is a S-shaped relationship between AC and NAFLD risk. NAFLD and AC < 34.5 cm show a statistically significant correlation. When AC is greater than 34.5 cm, its size has nothing to do with NAFLD, which may imply that AC has a dose-dependent effect on NAFLD risk.

Obesity leads to an elevation in the transportation of fatty acids from adipose tissue to various peripheral tissues (29). The increase of free fatty acids (FFA) derived from adipose tissue is mainly due to the resistance of adipose tissue to the anti-lipolysis effect of insulin (30). Extensive research findings strongly suggest that abnormally increased visceral fat serves as an indicator of excessive release of systemic FFAs (31). However, it is important to highlight that the majority of FFAs are released from the upper-body subcutaneous fat (32). Large AC means excessive subcutaneous fat accumulation, which helps release more fatty acids into the circulation. These circulating FFAs serve as crucial mediators in the development of metabolic disorders (33). It is worth noting that elevated plasma FFAs can trigger insulin resistance, promote inflammation, and lead to the synthesis and abnormal storage of triglycerides (34, 35). The excessive release of FFAs comes from the excessive accumulation of subcutaneous fat in the arms, which may be a potential mechanism partially explaining the correlation between AC and NAFLD.

Given the alarming rise in childhood obesity rates, it is crucial to prioritize efforts toward effectively managing the prevalence of NAFLD among children. Therefore, it is necessary to develop a standardized screening method to promote the early detection, diagnosis and treatment of NAFLD in pediatric medicine. By establishing clear screening and diagnostic criteria, we can provide clinicians with the necessary tools to identify and address NAFLD promptly. The role of AC in resource-constrained environments is crucial for clinicians to quickly and conveniently screen NAFLD. The precise exclusion of NAFLD in high-risk populations, such as obese children, enables clinicians to reduce the incidence of metabolic syndrome and other complications associated with NAFLD among children and adolescence.

There are several noteworthy advantages in this study. First of all, the measurement of hepatic steatosis in this study was conducted using VCTE, which has proven that it is more sensitive and accurate compared with ultrasound scanning (29). Secondly, the researchers utilized a large sample size of 1,559 children and adolescence from the NHANES database, ensuring the reliability of the study results. Despite these advantages, it is important to acknowledge the presence of several limitations. First of all, due to the cross-sectional of the study, the underlying mechanism between AC and NAFLD was not thoroughly investigated. Additionally, the diagnosis of hepatic steatosis in defining NAFLD relied on CAP values rather than liver biopsy. Furthermore, the influence of body fat was not evaluated in this study. Although AC was used as a convenient and practical method to evaluate subcutaneous arm fat, it was unable to quantify the extent of fat accumulation.

## Conclusion

In conclusion, higher AC levels are associated with an increased risk of NAFLD among American children and adolescence. AC can be regarded as a potential predictive indicator for NAFLD and NAFLD related liver steatosis.

## Data availability statement

Publicly available datasets were analyzed in this study. This data can be found at: www.cdc.gov/nchs/NHANEs/, National Health and Nutrition Examination Survey.

## Ethics statement

The studies involving humans were approved by the National Center for Health Statistics Ethics Review Board. The studies were conducted in accordance with the local legislation and institutional requirements. Written informed consent for participation in this study was provided by the participants’ legal guardians/next of kin.

## Author contributions

XW: Writing – original draft. JX: Methodology, Supervision, Writing – review & editing. SY: Writing – original draft, Writing – review & editing.

## Footnotes

^1^ http://www.empowerstats.com

^2^ http://www.R-project.org
